# Establishment of a Mouse Degenerative Model of Patellar Tendinopathy with Upregulation of Inflammation

**DOI:** 10.3390/ijms25073847

**Published:** 2024-03-29

**Authors:** Pauline Po Yee Lui, Zuru Liang, Ri Min Tan, Patrick Shu Hang Yung

**Affiliations:** Department of Orthopaedics and Traumatology, The Chinese University of Hong Kong, Shatin, New Territories, Hong Kong, China

**Keywords:** animal model, collagenase, patellar tendinopathy, inflammation

## Abstract

There is no mouse model of patellar tendinopathy. This study aimed to establish a mouse inflammatory and degenerative patellar tendon injury model, which will facilitate research on patellar tendinopathy using advanced molecular tools including transgenic models. Collagenase at different doses (low dose (LD), medium dose (MD), high dose (HD)) or saline was injected over the mouse patellar tendon. At weeks 1, 2, 4, and 8 post-injection, the tendons were harvested for histology and further examined by micro-computed tomography (microCT) imaging at week 8. The optimal dose group and the saline group were further evaluated by immunohistochemical staining, gait pattern, and biomechanical properties. The histopathological score increased dose-dependently post-collagenase injection. Ectopic mineralization was observed and increased with collagenase dose. The LD group was selected for further analysis. The expression of IL-10, TNF-α, and MMP-1 significantly increased post-injection. The changes of limb idleness index (ΔLII) compared to preinjury state were significantly higher, while the ultimate load, stiffness, ultimate stress, and maximum Young’s modulus were significantly lower in the LD group compared to the saline group. A mouse inflammatory degenerative model of patellar tendon injury resembling tendinopathy was established as indicated by the dose-dependent increase in tendon histopathology, ectopic calcification, decrease in biomechanical properties, and pain-associated gait changes.

## 1. Introduction

Patellar tendinopathy is an overuse tendon disorder characterized by activity-related tendon pain, swelling, and physical disability. It is common in sports such as soccer, basketball, and volleyball. About one in three male collegiate basketball players were reported to present with either patellar tendinopathy or patellar tendon abnormality [1]. The prevalence of patellar tendinopathies was 13.4% in the youth elite soccer players [2]. There is no effective treatment of patellar tendinopathy due to its unclear pathogenesis. Conservative treatment including physiotherapy, nonsteroid anti-inflammatory drugs, and steroids are not effective. The administration of steroids can also weaken the tendons [3,4]. The outcomes of surgical operation are unsatisfactory, with recurrent pain and tendon retear after surgery [5]. The pathogenic mechanisms of degenerative tendinopathy remain to be fully characterized. However, growing evidence suggests that excessive inflammation [6,7,8] and erroneous differentiation of tendon-derived stem/progenitor cells (TDSCs) [8,9,10] are key contributors to its progression. Tendon overuse initiates an inflammatory cascade that reduces the expression of tenocyte markers and increases the expression of nontenocyte markers in TDSCs, causing tissue metaplasia [8]. More TDSCs, but with a lower proliferative capacity and tenogenic potential, and higher cellular senescence, nontenocyte differentiation potential, and inflammatory response, were present in clinical samples and animal models of tendinopathy [8,10,11,12]. Tendinopathy TDSCs were unable to differentiate into tenocytes following mechanical stretch [10]. Diseased tendon stromal fibroblasts isolated from patients with tendinopathy exhibited more profound induction of inflammatory markers compared to healthy cells after IL-1β stimulation [12], a treatment commonly used to mimic overuse-induced inflammation in tendons [13,14,15]. The erroneous differentiation of TDSCs reduces the pool of TDSCs for tenogenesis and leads to failed healing.

Clinical samples of degenerative tendinopathy are useful for understanding the disease pathogenesis. However, they are usually obtained only at the late stage of the disease that requires surgery. The availability of animal models would facilitate the research of the disease pathogenesis and treatment. Both overuse- and collagenase-induced tendon injuries are frequently used for the development of tendinopathy animal models [16]. Rat, rabbit, dog, and horse models of tendinopathy were available for research [16]. Mice are frequently used for the development of animal models of various human diseases due to their short life cycle and availability of advanced tools such as transgenic animal models and antibodies. There is no mouse model of patellar tendinopathy, which impedes the research in this field. Tendinopathy is previously considered as a degenerative disease with an absence of infiltration of inflammatory cells in histology. Notwithstanding the argument, signs of inflammation were reported in clinical samples [6,7]. Inflammation is now recognized as a key component in the pathogenesis of tendinopathy and is a target for intervention. This study therefore aimed to establish a mouse model of inflammatory and degenerative patellar tendinopathy by collagenase injection, which will facilitate the study of molecular pathogenesis and treatment using advanced molecular tools. We hypothesized that a mouse patellar tendinopathy model that exhibits key histological and clinical characteristics of tendinopathy could be established. Histopathological changes of tendinopathy including hypercellularity, hypervascularity, loss of cell alignment, cell rounding and extracellular matrix (ECM) degeneration, fat infiltration, chondrocyte-like cells, and ectopic bone formation could be observed in this animal model. The injured tendon would also presented with an upregulation of inflammation. There would be a change in gait pattern due to tendon pain and a decrease in the biomechanical properties of the injured tendon.

## 2. Results

### 2.1. Histopathology

**Figure 1:**
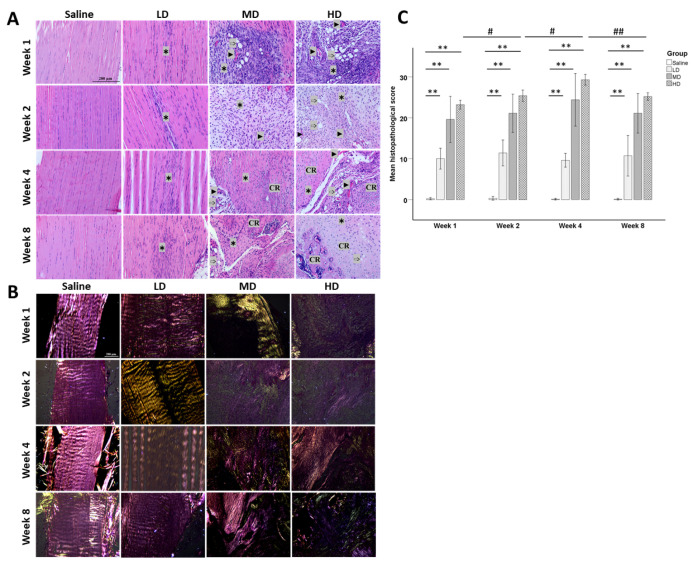
Histopathology of patellar tendons at different time points after saline or collagenase injection. (**A**) Photomicrographs showing the histopathological changes of the patellar tendons after injection with different concentrations of collagenases (LD: low dose; MD: medium dose; HD: high dose) at weeks 1, 2, 4, and 8. Scale bar: 200 µm; stain: haematoxylin and eosin; ✱: hypercellularity; ►: blood vessels; ➩: fat accumulation; CR: calcified region. (**B**) Polarized images of tendons. Scale bar: 200 µm. (**C**) Bar chart showing the mean histopathological scores of different groups at different time points. n = 5/time point/group; ** post hoc *p* < 0.01 compared to the saline group; # *p* < 0.05; ## *p* < 0.01 compared to different time points of the same group.

Saline injection had no effect on the histology of tendons and was similar to the histology of healthy tendons (Supplementary Material Figure S1). The tendon fibroblasts in the saline group were slender-shaped and longitudinally aligned between the parallel collagen fibers (Figure 1A). High collagen birefringence was observed under polarized microscopy. The injection of collagenase induced loss of collagen birefringence and the effect was dose-dependent (Figure 1B). Histopathological changes in tendons including loss of cell alignment, infiltration of inflammatory cells, hypercellularity, cell rounding, and collagen fiber disruption were observed as early as week 1 post-injection and maintained up to week 8. There was severe infiltration of inflammatory cells after collagenase injection in the medium dose (MD) and high dose (HD) groups and the number reduced with time after week 1. Hypervascularity, fat infiltration, chondrocyte-like cells indicated by round cells with lacunar space, and ectopic calcification were more commonly observed in the MD and HD groups. The chondrocyte-like cells were observed as early as week 2 and increased at week 4 in the MD and HD groups. Calcified region (CR) was observed in some samples in the MD group and all samples in the HD group at week 8 (Figure 1).

The histopathological score increased dose-dependently and was significantly higher in the collagenase groups compared to that in the saline group (all *p* < 0.05) (Figure 1C). There was no significant change of the histopathological score with time in the low dose (LD) and MD groups. The histopathological scores remained similar over time in the LD and MD groups while the score peaked at week 4 post-injury in the HD group and slightly decreased at week 8 post-injury.

### 2.2. Micro-Computed Tomography (microCT) Imaging

**Figure 2:**
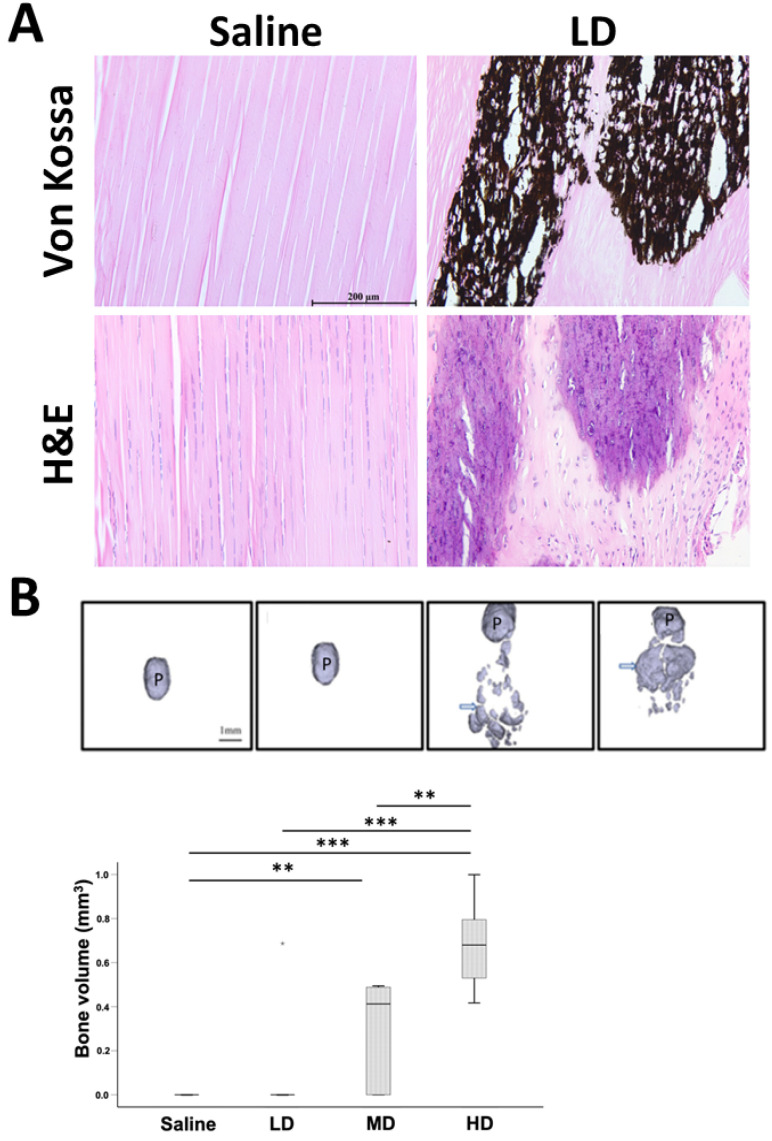
Evaluation of ectopic bone in tendon after collagenase injection. (**A**) Photomicrographs showing ectopic bone in tendon in the LD group at week 8 post-injection. Scale bar: 200 µm; stain: haematoxylin and eosin, von Kossa stain. (**B**) micro-computed tomography (microCT) images and boxplot showing bone volume (BV) of mineralized tissue inside the patellar tendon in different groups at week 8 post-injection. Scale bar: 1 mm; arrows: ectopic mineralized tissue; P: patellar; n = 10/group; ** post hoc *p* < 0.01; *** post hoc *p* < 0.001. “*” above the LD group represents extreme value of the dataset with the value of more than 3 box-lengths from the 75th percentile.

One (10%), six (60%), and ten (100%) patellar tendon samples showed calcification inside the patellar tendons at week 8 after collagenase injection, as shown by von Kossa staining (Figure 2A), and the bone volume (BV) was statistically significantly higher compared to the saline group for the MD (*p* < 0.01) and HD groups (*p* < 0.001) (Figure 2B). BV in the HD group was significantly higher than the LD group (*p* < 0.001) and MD group (*p* < 0.01) (Figure 2B). The BV in the MD was marginally insignificant compared to the LD group (*p* = 0.051).

### 2.3. Immunohistochemistry

**Figure 3:**
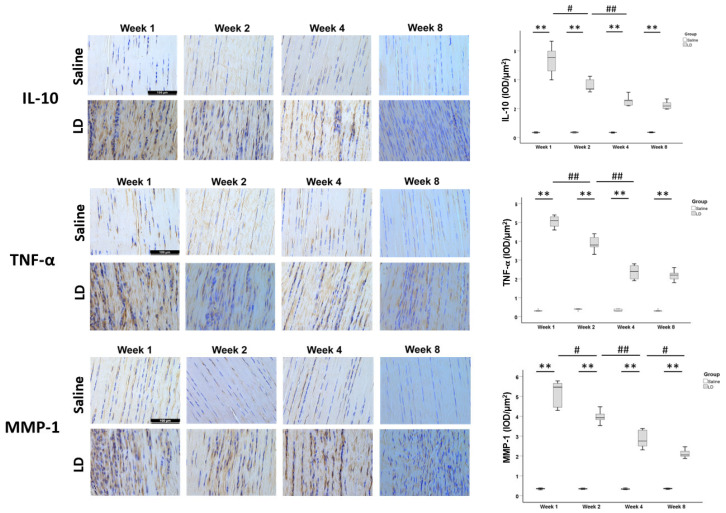
Expression of inflammatory cytokines and matrix-degrading enzyme in tendons after collagenase injection. Photomicrographs showing immunohistochemical staining of IL-10, TNF-α, and MMP-1 in the injured tendons at different times in the saline and LD groups. Scale bar: 100 µm. The boxplots show semiquantitative image analysis of MMP-1, IL-10, and TNF-α signal intensity (IOD/µm^2^) of the saline group and LD group at different time points. n = 5/time point/group; ** post hoc *p* < 0.01 compared to the saline group; # *p* < 0.05; ## *p* < 0.01 compared to different time points of the same group.

The expression of IL-10 and TNF-α at week 1 after saline injection was similar to that in healthy tendons (Supplementary Material Figure S2). Very weak expression of MMP-1 was observed in healthy tendons at week 1 and its expression was slightly higher in the saline group (Supplementary Material Figure S2). Weak expression of IL-10, TNF-α, and MMP-1 was observed in the tendon cells and the ECM in the saline group from week 1 to week 8 (Figure 3). The expression of IL-10, TNF-α, and MMP-1 increased significantly in the LD group starting from week 1 after injection, reduced with time, but remained higher compared to the saline group (*p* < 0.01 at all-time points) (Figure 3). The expression of these markers was observed mainly in the hypercellular regions and their surrounding ECM (Figure 3).

### 2.4. Gait Analysis

**Figure 4:**
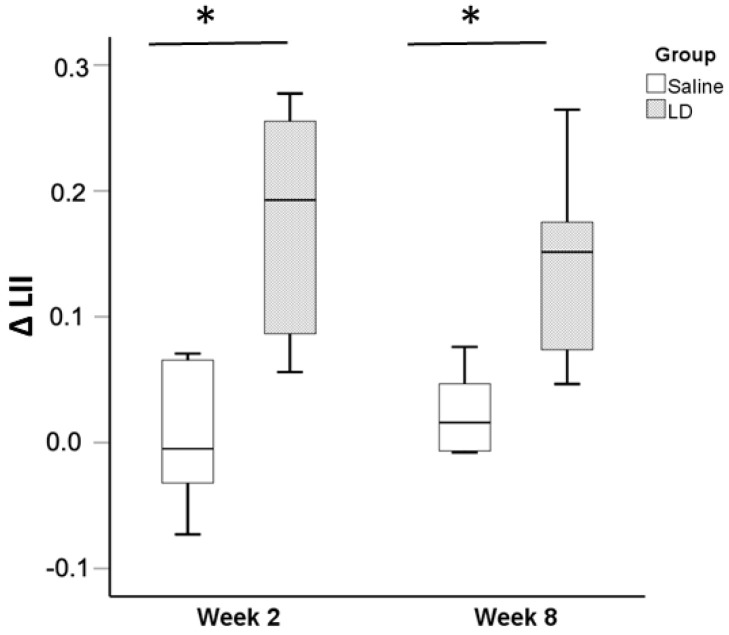
Changes of pain-associated gait pattern at week 2 and week 8 after collagenase injection. Boxplots showing changes of limb idleness index (ΔLII) compared to preinjury state in the saline and LD groups at week 2 and week 8 post-injection. n = 6/time point/group; * *p* < 0.05.

The changes of limb idleness index (ΔLII) compared to preinjury state was significantly higher in the LD group compared to the saline group at week 2 and week 8 post-injury (both *p* < 0.05) (Figure 4). There was no significant change in ΔLII from week 2 to week 8 in both groups (both *p >* 0.05) (Figure 4).

### 2.5. Biomechanical Test

**Figure 5:**
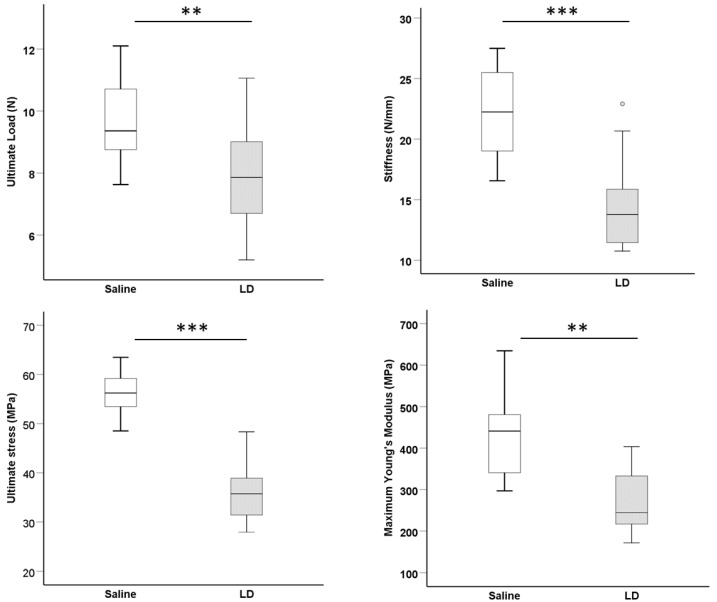
Biomechanical properties of the patellar–patellar tendon–tibia composite after injury. Boxplots showing ultimate load (N), stiffness (N/mm), ultimate stress (MPa), and maximum Young’s modulus (MPa) of the saline and LD groups at week 8 post-injection. n = 15/group; ** *p* < 0.01; *** *p* < 0.001. “o” above the LD group represents outliner of the dataset with the value of between 1.5 and 3 box-lengths from the 75th percentile.

At week 8 post-injury, the ultimate load (*p* < 0.01), stiffness (*p* < 0.001), ultimate stress (*p* < 0.01), and maximum Young’s modulus (*p* < 0.01) were significantly lower in the LD group compared to the saline group (Figure 5). Most of the samples failed at the patellar tendon to tibial junction and there was no difference in the failure mode between the saline group and LD group.

## 3. Discussion

Our results showed that collagenase injection dose-dependently induced matrix degeneration, collagen fiber disruption, hypercellularity, hypervascularity, cell rounding, fat accumulation, and ectopic calcification in the mouse patellar tendon. There were alterations in the pain-associated gait pattern, and the biomechanical properties of the injured patellar tendon was reduced after collagenase injection. The expression of IL-10, TNF-α, and MMP-1 significantly increased after collagenase injection, supporting activation of matrix degradation and inflammation in tendons after collagenase-induced tendon injury. IL-10 is an anti-inflammatory cytokine. The increased expression of IL-10 after collagenase injection might be explained by its attempt to suppress excessive inflammation in tendons after injury. The expression of IL-10, TNF-α, and MMP-1 in the injury groups decreased with time but remained higher than the expression in the saline group, suggesting that inflammation was reduced and might become chronic over time. Poor biomechanical properties of tendon, likely due to MMP production and matrix degeneration, might predispose to spontaneous tendon rupture, as observed in human tendinopathy. The results resemble the histopathology, radiological changes, and pain observed in tendinopathy. A degenerative mouse model of patellar tendon injury was successfully established. The sustained inflammation after collagenase-induced tendon injury in this animal model can be a target for intervention.

Animal models are a useful tool for studying the pathogenesis and treatment of tendinopathy before evaluation in patients. Animals are housed and treated under a standardized condition in each experiment, reducing random variation and increasing the reliability of the conclusion. As the etio-pathogenesis of chronic tendinopathy is multifactorial, there is no single animal model that can reproduce all the pathological changes of tendinopathy observed in patients [16]. Tendon overuse and intratendinous collagenase injection are two commonly used animal models of tendinopathy, each focusing on different processes of disease development. Tendon overuse aims to develop an animal model by introducing one possible cause of tendinopathy. The frequency and dose of tendon loading required to induce tendinopathy vary in different individuals, including animals. It showed variable success in recapitulating the pathological features of tendinopathy, as it can be difficult to force all rodents to run sufficiently fast or intensely to reach the level necessary for overuse. Animals may refuse to run on the treadmill. The overuse tendons recovered with rest within as short as 2 weeks after running for 2 or 4 weeks; hence, the model may not be reproducible and consistent with the defective healing response observed in human tendinopathy [17]. Some researchers included a run-in period to exclude the noncooperative animals to increase the reliability of the results. The time required for establishing the animal model is long and labor-intensive.

On the other hand, intratendinous collagenase injection targets replication of the degenerative changes in tendinopathy as collagenase induces damage to the ECM of tendon, with increased expression of MMPs and tissue inhibitor of matrix metalloproteinases (TIMPs), which are commonly observed in tendinopathy in patients [18,19,20]. Intratendinous collagenase injection in rat patellar tendon was reported to histologically induce hypercellularity [21], hypervascularity [21], matrix degeneration [21], change in matrix composition with sustained expression of proteoglycans and a high collagen type III/collagen type I ratio [22], and chondroid metaplasia [21]. There was also formation of ectopic mineralized tissue in microCT imaging [21], hypoecogenicity in ultrasound imaging, and pain-associated gait changes [23] in the rat collagenase-induced tendon injury model. The damaged tendon failed to heal up to week 32 after collagenase injection, reminiscent of failed healing in human tendinopathy [21]. Recent studies suggested that inflammation is a key component in the pathogenesis of tendinopathy [6,7,8]. Collagenase-induced tendon injury activated inflammatory response in the tendon [24,25], supporting that collagenase injection is a good model for mimicking the inflammatory and degenerative response in chronic tendinopathy. Tendon overuse might initiate an inflammatory event that subsides and then was overtaken by the degenerative changes in tendons [26]. Since the dose and site of administration of collagenase can be controlled, individual animal variation of tendon injury is reduced.

While there are collagenase-induced tendinopathy models in rats, rabbits, dogs, and horse [16], there have been very few studies using mouse models. The mouse genome is well characterized. Because of their shorter life span and small size, the cost for drug experiments is lower. Transgenic animal models and advanced molecular tools, assay kits, and antibodies are more readily available for mice. There have been only a handful of studies reporting the use of collagenase-induced mouse Achilles tendinopathy model [25,27,28,29]. However, the dose and volume of collagenase used appear too high [28]. We injected 0.2 mg of bacterial collagenase I in the mouse Achilles tendon and the tendon ruptured at week 1 after injection (unpublished results). There has been no mouse model of patellar tendinopathy. The mechanical loading of patellar tendon is different from the Achilles tendon and the patellar tendon is connected to two bony ends. The difference in anatomy and loading of Achilles and patellar tendons suggests the need to establish a site-specific mouse model for the study of pathogenesis and treatment of patellar tendinopathy.

We observed dose-dependent histopathological, radiological, and functional changes in tendon after collagenase injection. The exact dose of collagenase for the development of the animal model depends on the purpose of the study. For studies that aim to investigate the mechanisms and treatment of ectopic calcification, MD and HD treatment may be more appropriate as more calcified tissue was formed. However, not all human tendinopathy cases showed ectopic calcification. A total of 28.6% (8 out of 28 knees) [30] and 43.9% (18 out of 41 knees) [31] of patellar tendinopathy samples had calcification as confirmed by ultrasound imaging. LD treatment, therefore, might more closely reflect the prevalence of ectopic calcification of patellar tendinopathy.

Lipids, rounded chrondrocyte-like cells, and calcified tissue were observed in tendons after injection of collagenase in the mouse tendons, similar to the human samples of tendinopathy.

The mechanisms of tissue metaplasia might be due to erroneous differentiation of TDSCs [8,9,10] due to an increase in tendon inflammation in tendinopathy [6,7,8]. Tendon inflammation was reported after repetitive mechanical loading [15,32], which is modulated by other factors such as metabolic diseases, age, and drug used. Diseased tendon stromal fibroblasts isolated from patients with tendinopathy were found to exhibit more profound induction of inflammatory markers compared to healthy tendon stromal fibroblasts upon IL-1β treatment [12]. We also observed an increase in TNF-α expression in the mouse patellar tendons after collagenase injection. The tenogenic properties of tendon stem/progenitor cells were compromised in an inflammatory environment. For example, in situ tendon stem/progenitor cells underwent osteochondral differentiation rather than tenogenesis in an inflammatory niche in diseased tendons [8]. IL-1β, which is overexpressed in clinical samples of tendinopathy [13], reduced the expression of tenogenic markers but increased the expression of nontenogenic markers in TDSCs [33]. The unloading of tendon cells as a result of disruption of collagen fibers after collagenase injection or overuse-induced microtears of collagen fibers might also induce erroneous cell differentiation [34,35]. The disruption of collagen fibers providing tension to the resident tendon stem/progenitor cells by collagenase might induce unloading and erroneous cell differentiation. Further studies are required to validate the hypothesis.

Our study is novel as there is no similar mouse collagenase-induced degenerative patellar tendon injury model. This mouse model is particularly useful for studying the pathogenesis of patellar tendinopathy, especially with regard to the roles of inflammation in the disease. Transgenic animals with either overexpression or knockdown of target genes can be employed to elucidate the mechanisms involved. Additionally, the availability of antibodies only for mouse species provides a unique opportunity to study the protein’s potential roles in the disease pathogenesis. Furthermore, this animal model offers a valuable and cost-effective means of studying the impact of metabolic risk factors on disease severity and on drug treatment efficacy.

However, this study is not without limitations. First, the follow-up duration was short. Further research should examine the long-term effect of collagenase injection on tendons. Second, the mechanisms of collagenase injection in inducing ectopic mineralization, chondrocyte-like cells and fat accumulation, expression of inflammatory cytokines, and changes of gait pattern require further studies. Third, more inflammatory cytokines and matrix-remodeling markers should be examined.

## 4. Materials and Methods

### 4.1. Study Design

The animal research ethics committee and the University Laboratory Safety Office approved the study. The ARRIVE guidelines were followed.

**Figure 6:**
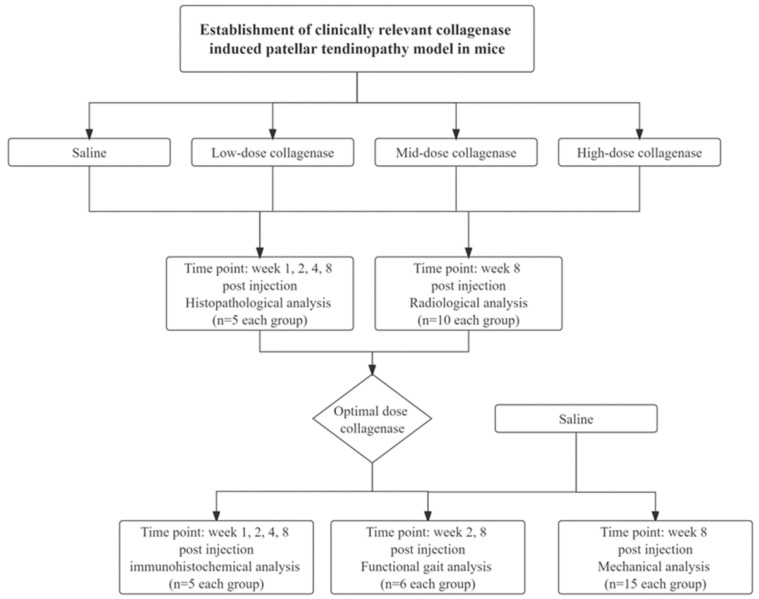
Overview of the study design.

A total of 150 male C57BL/6J mice (12-week-old, 25–29 g) were randomly divided into 4 groups—Group 1: saline; Group 2: low-dose collagenase (0.005 mg) (LD group); Group 3: medium-dose collagenase (0.015 mg) (MD group); and Group 4: high-dose collagenase (0.03 mg) (HD group). At weeks 1, 2, 4, and 8, the patellar tendons of one batch of animals were harvested for hematoxylin and eosin (H&E) or Von Kossa stain staining and histopathological scoring (n = 5/time point/group). At week 8, the ectopic bone (bone volume (BV), mm^3^) in tendons was assessed by microCT imaging (n = 10/group). Based on the histological and radiological findings, the optimal dose group was selected for further evaluation of the expression of inflammatory cytokines (IL-10 and TNF-α) and matrix degrading enzyme (MMP-1) (n = 5/group) by immunohistochemistry (IHC) and semiquantitative image analysis [36], pain-associated gait changes (n = 6/group) at week 2 and week 8 [23,37], and biomechanical properties of tendon (n = 15/group) at week 8 post-injection. Healthy patellar tendons were used as uninjured controls for comparing the histology and IHC results with the saline group at week 1 after injection. Figure 6 shows the study design. Male mice were used to reduce the effects of female hormones on the tendon pathology.

### 4.2. Collagenase-Induced Tendon Injury (CI Injury)

**Figure 7:**
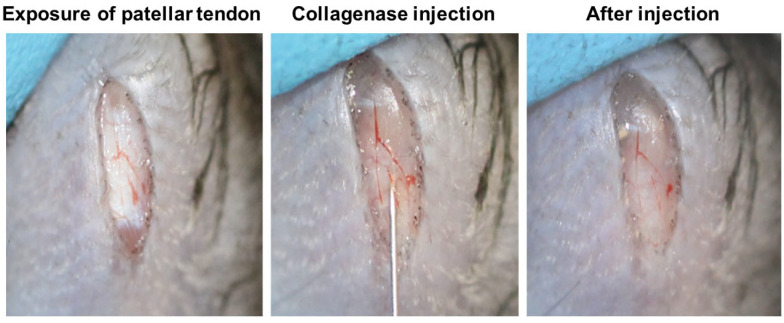
Photographs showing the surgical procedures of collagenase injection over the mouse patellar tendon. Briefly, a longitudinal skin incision was made and the patellar tendon was exposed. Bacterial collagenase I was then injected between the fascia and patellar tendon with a 32G needle. There was no leakage of collagenase after collagenase injection.

Fifteen minutes prior to aesthesia, the mice were administered with Temgesic (0.05 mg/kg) subcutaneously for pain relief. After anesthesia with intraperitoneal injection of ketamine (60 mg/kg, i.p.) and xylazine (4 mg/kg, i.p.), hair over the hind left limb was shaved. A 5 mm skin incision was made to expose the central region of the left patellar tendon with the knee flexed at 90°. A total of 5 µL (1 µg/µL, 3 µg/µL, or 6 µg/µL in 0.9% saline) of bacterial collagenase I (Sigma-Aldrich, St Louis, MO, USA) or saline was injected with a 32G needle (Hamilton, Timis County, Romania) over the middle region of the patellar tendon (Figure 7). The surgical wound was closed by 5.0 nylon sutures. The mice were kept under a 12 h light–dark cycle, 17–24 °C, and 70% humidity. They were given Temgesic (0.05 mg/kg) subcutaneously twice daily for the following 3 days post-surgery for pain relief and were allowed to have free cage activity immediately after surgery. Loss of body weight by 20% was taken as the humane endpoint.

### 4.3. Histology

Histology was performed according to an established protocol. Briefly, the patellar tendon was washed in phosphate-buffered saline (PBS), fixed in 10% buffered formalin, and embedded in paraffin. Five-micrometer-thick coronal sections in the middle of the patellar tendon were cut and mounted onto coated slides and stained with H&E or Von Kossa stain for ectopic bone if necessary and examined under light microscopy (DM5500B, Leica Microsystems GmbH, Wetzlar, Germany). One slide in the middle of each patellar tendon was used for histological evaluation. Loss of collagen fiber alignment was assessed under polarized light. Representative histological images were presented.

The histopathological changes were assessed using a ten-parameter scoring system adapted and modified based on our established scoring system for the assessment of healing of rat patellar tendons. The scoring system assesses tendon histopathology based on (1) fiber arrangement; (2) cellularity; (3) cell alignment; (4) cell rounding; (5) vascularity; (6) fiber structure; (7) hyaline degeneration; (8) inflammation; (9) calcification; and (10) fat accumulation. Each parameter has a score of 0–3, with 0 indicating no histopathological changes and 3 indicating the most severe histopathological changes. The histopathological score was calculated by summing the scores of each parameter. Therefore, a healthy tendon should have a score of 0 while a tendon with maximal histopathology should have a score of 30. The scoring system has high intrarater reliability with intraclass correlation (ICC) of 0.992 (0.979–0.997) (all *p* < 0.001) and high interrater reliability with ICC of 0.982 (0.852–0.993) (all *p* < 0.001) for the 10 parameters, and the results were considered to be excellent.

### 4.4. MicroCT Imaging

Ectopic mineralization in tendons was examined with the use of a cone-beam microCT system (µCT40, Scanco Medical, Brüttisellen, Switzerland) with voxel size of 30 µm^3^. The patellar tendon samples were scanned with the patella and the tibial plateau as the landmarks. The images were then 3D-reconstructed after thresholding, using the built-in software. The patellar tendon-only region was selected as the region of interest (ROI). The bone volume (BV) (mm^3^) inside the patellar tendon was measured using the system software.

### 4.5. Immunohistochemistry

Immunohistochemical staining of inflammatory markers (IL-10, TNF-α) and matrix-degrading enzyme (MMP-1) was performed as described previously. After deparaffination, the sections were rehydrated, decalcified, quenched with endogenous peroxidase activity with 3% hydrogen peroxide for 15 min, and treated with 10 mM citrate buffer at 70 °C for 1.5 h. After blocking with 1% BSA in PBS, the sections were incubated with specific antibodies against IL-10 (dilution: 1:200; Abcam, Cambridge, UK; catalog # ab217941), TNF-α (dilution: 1:200; Abcam; catalog # ab6671) or MMP-1 (dilution: 1:200; Proteintech, IL, USA; catalog # 10371-2-AP) diluted in 1% BSA at 4 °C overnight. After washing with PBS, the sections were incubated with horseradish peroxidase (HRP)-conjugated secondary antibody (1:500; Thermo Fisher Scientific Inc., Massachusetts, USA; catalog # 31466) for 1 h at room temperature. The 3.3′Diaminobenzidine (DAB, Thermo Fisher Scientific Inc.; catalog # 34002) was used for color development. The sections were then rinsed, counterstained with hematoxylin, dehydrated with graded ethanol and xylene, and mounted with p-xylene-bis-pyridinium bromide (DPX) permount (Sigma Aldrich, St Louis, MO, USA). The primary antibody was replaced with 1% bovine serum albumin (BSA) in the negative controls. All the incubation times and conditions were strictly controlled. The samples from different groups were stained in the same batch. The sections were examined under a light microscope (DMRXA2, Leica Microsystems GmbH, Wetzlar, Germany). Two fields of view at 200× magnification of each sample were taken for image analysis. The immunopositive signal was quantified and presented as integrated optical density (IOD) of region of interest (IOD/µm^2^) using the Image Pro Plus 9.0 software (Media Cybernetics, Bethesda, MD, USA).

### 4.6. Gait Analysis

Gait analysis was performed longitudinally before collagenase injection and then at weeks 2 and 8 using the Catwalk XT 9.0 system (Noldus Information Technology, Wageningen, The Netherlands) according to our established protocol. Briefly, the mouse was habituated to the CatWalk and allowed to walk ad libitum across the glass walkway. The following day, the mouse was tested by crossing the glass walkway for three compliant runs. A compliant run is defined as a mouse walking across the glass walkway without stopping, turning around, or changing direction, and with less than 30% speed variation. The region of interest (ROI) (5 × 30 cm) for image capture was defined and calibrated. Two infrared light beams spaced 60 cm apart were used to detect the arrival of the mouse and control the start and end of data acquisition. The footprint was recorded by a high-speed video camera that was positioned 34 cm away under the glass walkway. Footprints were automatically captured when the mouse entered the defined walkway and were classified as the left forelimb, right forelimb, left hind (LH) limb, and right hind (RH) limb by the built-in software. At preinjury and weeks 2 and 8 after injection, pain-related gait changes in mice (n = 6 per group) were assessed by the catwalk system. The Limb Idleness Index (LII) is a validated measure of pain-associated gait changes at the knee [37]. It was calculated as a product of anchor print ratio, target print ratio, and swing duration ratio, as described previously [37]. The difference (Δ) of LII at the time of post-injury compared to preinjury state was calculated and compared between groups. An increase in ΔLII suggested pain-associated gait changes.

### 4.7. Ultrasound Imaging

The knee samples were scanned using the animal ultrasound system (Vevo LAZR, FUJIFILM VisualSonics, Toronto, ON, Canada) before the mechanical test. The ultrasound system is equipped with a handling platform, a 3D motor, and a Transducer MS700 (30–70 MHz). Briefly, the knee was thawed to room temperature, and then shaved and fixed by a rubber clay on the handling platform in a supine position with knee flexion of approximately 90°. Coupling gel was added to cover the knee. The transducer was placed over the knee, aligned parallel to the patellar tendon, and scanned from the medial to the lateral side. Then, 3D ultrasound images were collected for 3 mm with a step size of 0.025 mm. The total volume and length of the tendon were measured using the system software. The cross-sectional area was calculated by dividing the total volume by the length of the tendon.

### 4.8. Biomechanical Test

The bone–patellar tendon–bone composite was isolated after ultrasound imaging. The sample was kept moist with 0.9% saline during the experiment. The composite was fixed onto a tensile testing machine with two clamps (Instron Electropuls E3000, High Wycombe, Bucks HP12 3SY, UK), with the proximal patellar secured by the upper clamp and the shaft and tibial plateau fixed by the lower clamp. A piece of sandpaper was placed between the sample and the clamp to increase friction. The sample was preloaded to 0.02 N. After preconditioning (5 cycles, maximal displacement of 0.1 mm, 0.03 mm/s), the sample was pulled to failure at a testing speed of 0.3 mm/s using a 50 N load cell. The load–displacement curve and the failure mode of the injured tendon tissue were recorded. The ultimate load (N) was read by the load–displacement curve. The ultimate stress (MPa) was calculated as the ultimate load divided by the tendon cross-sectional area. The stiffness (N/mm) was the slope of the linear region at 35–60% of load-displacement curve. The maximum Young’s modulus (MPa) was calculated from the linear slope of the stress–strain curve. The second author performed all the assessments, and the same assessment for different groups at the same time points were performed on the same day to reduce bias. Statistical data analysis was performed by the first author, who was not involved in the assessment.

### 4.9. Sample Size Calculation

Ultimate stress at week 8 was used as the primary outcome for sample size calculation. The ultimate stress of the saline group was 56.0 ± 4.4 MPa. At α = 0.05; 1 − β = 0.90, 15 animals per group were required to detect 10% decrease in ultimate stress, which was clinically significant.

### 4.10. Data Analysis

All animals were included in data analyses. The quantitative and semiquantitative data are presented in boxplots or bar charts. For data presented in boxplots, the box length represents the difference between 25th and 75th percentiles. The horizontal line inside the box represents the median. The whiskers represent the largest and lowest values of the dataset that are not outliners or extreme values. The “o” outside the box represents outlier with the value between 1.5 and 3 box-lengths from the 75th percentile or 25th percentile. “*” represents extreme value with the value more than 3 box-lengths from the 75th percentile or 25th percentile. The bar chart shows the mean ± standard deviation (SD) of the dataset. The comparison of two independent groups was performed by Mann-Whitney U-test The comparison of more than two independent groups was performed by means of the Kruskal–Wallis test, followed by post hoc pairwise comparison with the use of the Mann–Whitney U-test. ΔLII was compared between groups using Mann–Whitney U-test and between week 2 and week 8 using Wilcoxon signed-rank test. All the data analysis was performed using SPSS software (version 26.0). *p* < 0.05 was regarded as statistically significant.

## 5. Conclusions

In conclusion, a mouse degenerative model of patellar tendon injury resembling tendinopathy with an upregulation of inflammation was established after collagenase injection, as indicated by the dose-dependent increase in tendon histopathology, ectopic calcification, decrease in biomechanical properties, and pain-associated gait changes.

## Data Availability

All the data are included in the manuscript and available on request.

## Supplementary Materials

The following supporting information can be downloaded at: https://www.mdpi.com/article/10.3390/ijms25073847/s1.
