# Supplementary material for: Establishment of a Mouse Degenerative Model of Patellar Tendinopathy with Upregulation of Inflammation
Source: Int J Mol Sci. 2024 Mar 29;25(7):3847. doi: 10.3390/ijms25073847 (PMC11011606; doi:10.3390/ijms25073847)
Supplement: Supplementary file 1 [file ijms-25-03847-s001.zip › ijms-2861924-supplementary.pdf]

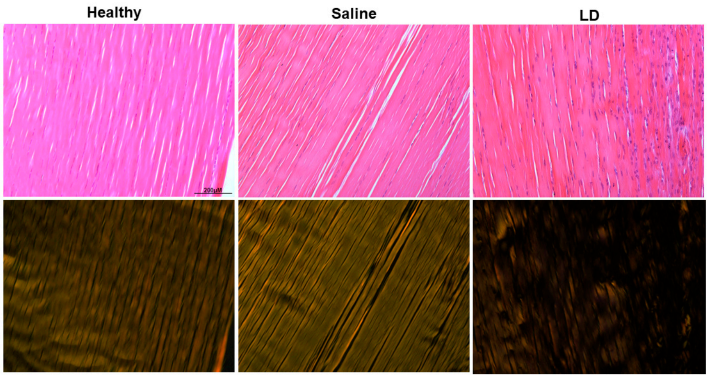

Supplementary material Figure S1. Photomicrographs showing histology and corresponding polarized images of healthy tendons, tendons in the saline group and LD group at week 1 after injection. Scale bar: 200 µm; stain: haematoxylin and eosin; n=4/group

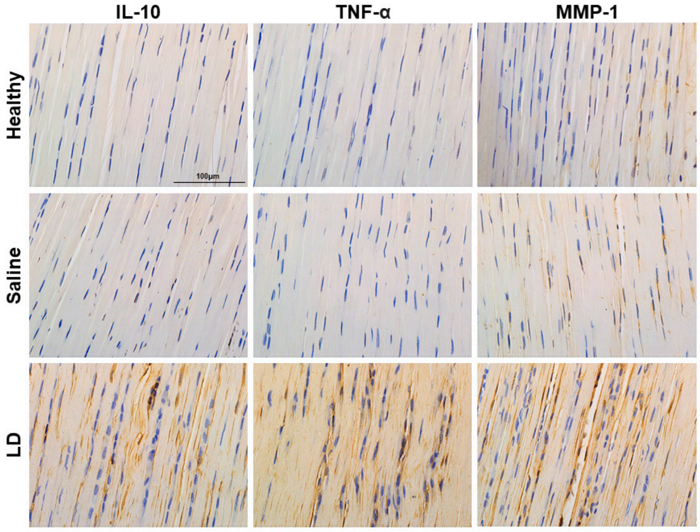

Supplementary material Figure S2. Photomicrographs showing immunohistochemical staining of IL-10; TNF-α; and MMP-1 in healthy tendons, tendons in the saline group and LD group at week 1 after injection. Scale bar: 100 µm; n=4/group
